# Antimicrobial Evaluation of Bacterial Isolates from Urine Specimen of Patients with Complaints of Urinary Tract Infections in Awka, Nigeria

**DOI:** 10.1155/2016/9740273

**Published:** 2016-04-20

**Authors:** Perpetua A. Ekwealor, Malachy C. Ugwu, Ifeanyi Ezeobi, George Amalukwe, Belinda C. Ugwu, Ugochukwu Okezie, Catherine Stanley, Charles Esimone

**Affiliations:** ^1^Department of Pharmaceutical Microbiology and Biotechnology, Faculty of Pharmaceutical Sciences, Nnamdi Azikiwe University, Agulu Campus, Awka, Nigeria; ^2^Anambra State University Teaching Hospital, Amaku, Awka, Nigeria; ^3^Department of Pharmaceutical Microbiology, Faculty of Pharmacy, University of Port Harcourt, Rivers State, Nigeria

## Abstract

Urinary tract infections (UTIs) account for one of the major reasons for most hospital visits and the determination of the antimicrobial susceptibility patterns of uropathogens will help to guide physicians on the best choice of antibiotics to recommend to affected patients. This study is designed to isolate, characterize, and determine the antimicrobial susceptibility patterns of the pathogens associated with UTI in Anambra State Teaching Hospital, Amaku, Anambra State, Nigeria. Clean catch urine samples of inpatient and outpatient cases of UTI were collected and bacteriologically analyzed using standard microbiological procedures. Antibiogram was done by the Kirby-Bauer disc diffusion method. The most prevalent isolates were* S. aureus* (28%),* E*.* coli* (24.6%), and* S. saprophyticus* (20%). The antibacterial activities of the tested agents were in the order of Augmentin < Ceftazidime < Cefuroxime < Cefixime < Gentamicin < Ofloxacin < Ciprofloxacin < Nitrofurantoin. It was found that all the organisms were susceptible in varying degrees to Nitrofurantoin, Ciprofloxacin, and Ofloxacin. It was also observed that all the bacterial species except* Streptococcus* spp. have a Multiple Antibiotic Resistance Index (MARI) greater than 0.2. For empiric treatment of UTIs in Awka locality, Nitrofurantoin, Ciprofloxacin, and Ofloxacin are the first line of choice.

## 1. Introduction

Urinary tract infection (UTI) is described as a bacteriuria with urinary symptoms [1]. It is one of the most common bacterial infections seen in clinical practice particularly in developing countries with a high rate of morbidity and financial cost [1]. Some of the key factors predisposing to urinary tract infection have been attributed to poor personal hygiene and urinary tract abnormalities [2–4]. The causative agents for urinary tract infection vary from place to place and they also vary in their susceptibility and resistance patterns. UTIs are caused by different microbial pathogens [4, 5]. The most common pathogenic organisms of UTI are* Escherichia coli, Staphylococcus saprophyticus, S. aureus*,* Proteus* sp.,* Klebsiella pneumoniae, Pseudomonas aeruginosa, *and enterococci [6–8]. Treatment of UTI cases is often started empirically and therapy is based on information determined from the antimicrobial resistance pattern of the urinary pathogens [9, 10]. In spite of the availability and use of the antimicrobial drugs, UTIs caused by bacteria have been showing increasing trends in recent years. Much of the increase has been related to emerging antibiotic resistance among urinary tract pathogens. Increasing multidrug resistance in bacterial uropathogens is an important and evolving public health challenge [1, 10]. The prevalence of antimicrobial resistance in urinary pathogens is increasing worldwide [11]. Accurate bacteriologic records of culture results provide guidance on empirical therapy before sensitivity patterns are available [11, 12]. Since most UTIs are treated empirically, the criteria for the selection of antimicrobial agents should be determined on the basis of the most likely pathogen and its expected resistance pattern in a geographic area [1, 13]. Thus, there is a need for periodic monitoring of causative agents of UTI and their resistance pattern in a given locality [1]. The resistance pattern of community acquired uropathogens has not been extensively studied in Awka. To date, no data regarding the bacterial resistance in UTIs from Amaku (a teaching hospital in the urban area of Awka city in Anambra State, southeastern Nigeria) has been documented. This study is, therefore, designed to determine the bacterial uropathogens and their antibiotic sensitivity patterns among patients with complaints of UTIs in Awka city.

## 2. Methods

### 2.1. Study Area

Clean catch urine samples were collected from both inpatients and outpatients of the Anambra State University Teaching Hospital, Amaku, Awka. This sample collection site was chosen because it covers the urban area of the city. The duration of the study was 4 months (April to August 2015). The study was carried out in the Microbiology Laboratory of the Department of Pharmaceutical Microbiology and Biotechnology, Faculty of Pharmaceutical Sciences, Nnamdi Azikiwe University, Agulu Campus, Awka.

### 2.2. Sample Collection/Study Design

The sample collection approach used was the clean catch method to minimize contamination [4]. Clean catch midstream urine was collected from each patient into a 20 mL calibrated sterile screw-capped universal container which was initially distributed to the patients. The specimen was appropriately labeled, transported to the laboratory, and analyzed within 2 hours after collection. Prior to sample collection, all patients were well instructed on how to collect the urine sample aseptically to avoid contamination. More so, verbal informed consent was obtained from all patients prior to specimen collection and the study was conducted after obtaining due ethical approval from the hospital administration (COOUTH/AA/VOI.I.002).

### 2.3. Sample Processing/Culture and Identification of Organisms

The samples were processed according to a previously described methodology [4]. Only patients that presented with clinical symptoms of UTI and positive urine culture (≥10^5^ CFU/mL) were studied. Briefly, each urine sample was aseptically inoculated (in triplicate) into MacConkey agar plates, mannitol salt agar plates, and cetrimide agar plates on arrival at the laboratory. The plates were incubated aerobically at 37°C for 18–24 hr. The characteristic bacterial isolates observed on the selective media plates were aseptically subcultured onto freshly prepared culture media plates; and the resulting cultures/isolates were subjected to microscopical and appropriate biochemical tests for proper identification. Identification of bacterial isolates was done on the basis of their cultural and biochemical characteristics as described in the notable book of Cheesbrough [14]. The identified bacterial isolates were maintained in nutrient agar slants, incubated at 37°C for 24 hr, and subcultured periodically.

### 2.4. Antibiotic Susceptibility Study

Antibiotic susceptibility patterns of the bacterialisolates were evaluated using disk diffusion assay [15]. The antibiotic disc (ABTEK, India) containing the following antibiotics was used: Ceftazidime (CAZ) 30 *µ*g, Cefuroxime (CRX) 30 *µ*g, Gentamicin (GEN) 10 *µ*g, Cefixime (CXM) 5 *µ*g, Ofloxacin (OFL) 5 *µ*g, Augmentin (AUG) 30 *µ*g, Nitrofurantoin (NIT) 30 *μ*g, and Ciprofloxacin (CPR) 5 *µ*g. Standardized overnight culture of each isolate was used to seed melted Mueller-Hinton agar (MHA) at 45°C and poured into sterilized plates (in triplicate) aseptically. These were allowed to solidify and the antibiotic disks were aseptically placed on the surface of the culture media. The MHA plates were then incubated at 37°C for 24 h. After 24 h incubation, the inhibition zones were measured and interpreted by the recommendations of the Clinical Laboratory Standards Institute (CLSI) [16].

### 2.5. Multiple Antibiotic Resistance Indices (MARI)

The MARI calculation was done by dividing the number of antibiotics to which a microorganism is resistant by the total number of antibiotics to which the organism was subjected.

## 3. Results and Discussion

Urinary tract infections (UTIs) are one of the most common bacterial infections in the human urinary system. They are mostly treated empirically and the criteria for the selection of antimicrobial agents should be determined on the basis of the most likely pathogen and its expected resistance pattern in the locality [1, 13]. Thus, there is a need for periodic monitoring of the causative agents of UTI and their resistance/susceptibility pattern in a locality.  Table 1 shows the details of culture characterization. Of the 100 positive urine cultures, 215 bacterial isolates were obtained.* S. aureus* was found to be the predominant and most frequently isolated urinary pathogen, followed by* E. coli*, and this was followed by* S. saprophyticus*, a coagulase-negative* Staphylococcus *species. Similar studies in south-southern Nigeria have previously reported that* S. aureus* was the predominant isolated uropathogen from patients with signs and symptoms of UTI [17]. The high proportion of* S. aureus* was attributed to receptive anal intercourse and HIV infections [17–19]. Some studies had previously linked the increasing cause of UTIs by* Staphylococcus* to increased use of instrumentation such as bladder catheterization [20, 21]. However, the observed high proportion of* Staphylococcus* varied with some previously published studies [1, 12, 22] where* E. coli* was found to be the predominant urinary tract pathogen. This variation further supports the fact that the distribution of UTI-causing pathogens, including their antimicrobial susceptibility pattern, varies from place to place and changes from time to time [23].* S. saprophyticus* has been reported as the second most frequently cultured uropathogen while streptococci are not frequently isolated but remain to be clinically important uropathogens [24]. This further supports the low level of* Streptococcus* spp. recorded in our study. In 2011, Burckhardt and Zimmermann admitted that published reports of UTI associated with streptococci are scarce but they maintained that streptococci are potential UTI-causing pathogens especially in subjects with urinary tract abnormalities [25]. The spectrum of Group B* Streptococcus* UTI includes asymptomatic bacteriuria (ABU), cystitis, pyelonephritis, urethritis, and urosepsis [26]. It should however be noted that the presence of* Bacillus* and* N. gonorrhoeae* in our study could be attributed to colonization and/or coinfection. Weigler et al. [27] showed that 18% of sexually active women presenting with urinary tract infection complaints were found to have an STD. They concluded that a wide variety of different STDs can be present in patients with UTI symptoms.

Considering the overall sensitivity of the isolates to the whole antibiotics tested as in Table 2, one could infer that* Streptococcus *sp. is the most sensitive isolate in this study, having good sensitivity to all the antibiotics tested. It was also found that all the organisms were well susceptible in varying degrees to Nitrofurantoin, Ciprofloxacin, and Ofloxacin. This is in line with previously published studies [1, 12, 13]. They all reported that Nitrofurantoin showed the best activity against all the isolates. Ciprofloxacin had previously been reported to be very active against uropathogens [4, 28]. Our predominant isolates (*S. aureus*,* E. coli*, and* S. saprophyticus*) showed variable resistance to most drugs used, including Amoxicillin-Clavulanic Acid, Cefuroxime, and Ceftazidime, even Cefixime to some extent. This finding is in line with the work of Uwaezuoke and Ogbulie [13]. Since a greater percentage of the UTI isolates in this study were sensitive to Nitrofurantoin, it would be an excellent choice for UTI empiric therapy while awaiting the result of culture and sensitivity tests. However, the patient's status may warrant the choice of Ciprofloxacin or Ofloxacin. Strikingly, the majority of the isolates were resistant to Amoxicillin-Clavulanic Acid. This high level of resistance observed with Amoxicillin-Clavulanic Acid can be attributed to the irrational use of drug in this locality. The increasing level of abuse of drugs by the public, where patients indulge in antibiotic self-medication, commonly to treat all kinds of infections, has been recorded as one significant way of promoting antibiotic resistance [29, 30]. Clavulanic Acid present in the Amoxicillin-Clavulanic Acid complex is meant to afford protection to the *β*-lactam chemical ring nucleus present in the Amoxicillin, and this protection should be expected to enhance the activity of Amoxicillin. Hence, the Amoxicillin-Clavulanic Acid complex should demonstrate clearly significant susceptibility rates over the isolates. This observed resistance is related to permeability and absorption factors influencing antibiotic transfer across the microbial cells. Thus, the Amoxicillin-Clavulanic Acid complex being a large molecule possibly would experience great difficulty in permeability and overall transport across the microbial cell wall [31]. As a result, high resistance may be due to the relatively limited quantity available to exert an antimicrobial effect [31, 32]. Our finding about the *β*-lactams especially the Cephalosporins correlates with the findings of Prakash and Saxena [1].

The result of the MARI is shown in Table 3. From the MARI obtained in this research, only one isolate gave MARI of < 0.20 and that was the* Streptococcus* spp. (0.125). Others gave higher MARI. MARI is a tool that reveals the spread of bacteria resistance in a given population [30, 31]. Any MARI greater than 0.20 implies that the strains of such bacteria originate from an environment where several antibiotics are used or misused [4, 32, 33]. This implies that a very large proportion of the bacterial isolates have been exposed to several antibiotics and thus have developed resistance to these antibiotics. Similar incidence was reported in the work of Ehinmidu (2003) [4] though not exactly with the same set of antibiotics. Prakash and Saxena [1] equally reported that uropathogens are resistant to commonly used antibacterials. The shortcoming of the study is that the patients' clinical data (age, gender, catheter-associated UTI, etc.) were not documented.

## 4. Conclusions

*S. aureus, E. coli*, and* S. saprophyticus* were the most prevalent among the uropathogens investigated. The high MARI from urine samples isolates in this locality underscores the need for continuous monitoring of antibiotic susceptibility profile of bacteria implicated in UTI prior to antibiotic prescription in order to ensure optimal and desired treatment. However, we recommended that, for empiric treatment of UTIs in Awka locality, Nitrofurantoin, Ciprofloxacin, and Ofloxacin are the first line of choice.

## Figures and Tables

**Table 1:** Incidence of bacterial isolates from urine samples of the patients.

Bacteria isolates	Number of isolates	Incidence rate (%)
*Staphylococcus aureus*	60	28
*Escherichia coli*	53	24.6
*Staphylococcus saprophyticus*	43	20
*Pseudomonas aeruginosa*	18	8.4
*Proteus *spp.	11	5.1
*Enterococcus faecalis*	10	4.6
*Klebsiella pneumoniae*	8	3.7
*Streptococcus *spp.	6	2.8
*Neisseria gonorrhoeae*	3	1.4
*Bacillus *spp.	3	1.4
Total	**215**	**100%**

**Table 2:** Antimicrobial susceptibility pattern of the bacterial isolates from urine specimens.

Isolates	Antibiotics																							
	Cefuroxime			Cefixime			Ofloxacin			Gentamicin			Amoxicillin-Clavulanic Acid			Nitrofurantoin			Ciprofloxacin			Ceftazidime		
	S	R	MS	S	R	MS	S	R	MS	S	R	MS	S	R	MS	S	R	MS	S	R	MS	S	R	MS
*S. aureus*	13.8	65.5	20.7	34.5	38	27.5	51.7	41.4	6.9	55.2	34.5	10.3	10.3	82.8	6.9	86.2	6.9	7.0	58.6	38	3.4	7	75.8	17.2
*S. saprophyticus*	4.7	81	14.3	19.0	47.6	33.3	81	14.3	4.7	28.6	66.7	4.7	9.5	71.4	19.0	90.5	4.7	4.8	66.7	19.0	14.3	4.8	76.2	19.0
*E. coli*	0	66.7	33.3	66.7	22.2	11.1	72.2	27.8	0	55.6	16.7	27.8	0	94.4	5.6	100	0	0	72.2	27.8	0	0	61.1	38.9
*E. faecalis*	0	70	30	60	40	0	40	50	10	60	40	0	0	90	10	80	10	10	60	40	0	0	90	10
*P. aeruginosa*	0	57	43	0	43	57	71.4	28.6	0	28.6	71.4	0	14.3	85.7	0	85.7	0	14.3	71.4	28.6	0	14.3	71.4	14.3
*Proteus *spp.	0	100	0	66.7	0	33.3	33.3	66.7	0	66.7	0	33.3	0	100	0	100	0	0	66.7	33.3	0	0	66.7	33.3
*Streptococcus *spp.	0	16.7	83.3	83.3	16.7	0	83.3	16.7	0	83.3	16.7	0	0	100	0	100	0	0	100	0	0	33.3	50	16.7
*K. pneumoniae*	0	66.7	33.3	66.7	33.3	0	100	0	0	66.7	0	33.3	0	66.7	33.3	66.7	0	33.3	100	0	0	0	66.7	33.3
*Bacillus*	0	33.3	66.7	0	33.3	66.7	66.7	33.3	0	66.7	33.3	0	0	66.7	33.3	66.7	0	33.3	66.7	0	33.3	0	33.3	66.7

S: % susceptible.

R: % resistant.

MS: % moderately sensitive.

**Table 3:** Multiple Antibiotic Resistance Indices (MARI) of the bacterial isolates.

Isolates	MARI	Antibiotics to which the isolates are resistant
*S. aureus*	0.500	CAZ, CRX, CXM, and AUG
*S. saprophyticus*	0.625	CAZ, CRX, CXM, GEN, and AUG
*E. coli*	0.375	CAZ, CRX, and AUG
*E. faecalis*	0.500	CAZ, CRX, OFL, and AUG
*P. aeruginosa*	0.500	CAZ, CRX, GEN, and AUG
*Proteus *spp.	0.500	CAZ, CRX, OFL, and AUG
*Streptococcus *spp.	0.125	AUG
*K. pneumoniae*	0.375	CAZ, CRX, and AUG
*Bacillus*	0.250	CAZ and AUG

Total number of antibiotics tested = 8.

CAZ: Ceftazidime; CXM: Cefixime; CRX: Cefuroxime; AUG: Amoxicillin-Clavulanic Acid; OFL: Ofloxacin; GEN: Gentamicin; NIT: Nitrofurantoin; CPR: Ciprofloxacin.
